# The *Candida albicans* Ku70 Modulates Telomere Length and Structure by Regulating Both Telomerase and Recombination

**DOI:** 10.1371/journal.pone.0023732

**Published:** 2011-08-23

**Authors:** Lidia Chico, Toni Ciudad, Min Hsu, Neal F. Lue, Germán Larriba

**Affiliations:** 1 Microbiology, Department of Biomedical Sciences, Faculty of Sciences, University of Extremadura, Badajoz, Spain; 2 Department of Microbiology and Immunology, W. R. Hearst Microbiology Research Center, Weill Medical College of Cornell University, New York, New York, United States of America; University of Minnesota, United States of America

## Abstract

The heterodimeric Ku complex has been shown to participate in DNA repair and telomere regulation in a variety of organisms. Here we report a detailed characterization of the function of Ku70 in the diploid fungal pathogen *Candida albicans*. Both *ku70* heterozygous and homozygous deletion mutants have a wild-type colony and cellular morphology, and are not sensitive to MMS or UV light. Interestingly, we observed complex effects of *KU70* gene dosage on telomere lengths, with the *KU70/ku70* heterozygotes exhibiting slightly shorter telomeres, and the *ku70* null strain exhibiting long and heterogeneous telomeres. Analysis of combination mutants suggests that the telomere elongation in the *ku70* null mutant is due mostly to unregulated telomerase action. In addition, elevated levels of extrachromosomal telomeric circles were detected in the null mutant, consistent with activation of aberrant telomeric recombination. Altogether, our observations point to multiple mechanisms of the Ku complex in telomerase regulation and telomere protection in *C. albicans*, and reveal interesting similarities and differences in the mechanisms of the Ku complex in disparate systems.

## Introduction

*Candida albicans*, the most common fungal pathogen for humans, exhibits a number of unusual biological characteristics, including obligate diploidy, a high level of polymorphisms in its genome, and a predominantly clonal mode of reproduction [1]. In the absence of meiosis, the genetic variability of the species is primarily derived from shuffling of the highly polymorphic parental chromosomes during the parasexual cycle as well as from mitotic recombination events occurring during both the parasexual and the mitotic cycles [2], [3]. There are two general pathways of recombination, homologous recombination (HR) and non-homologous end-joining (NHEJ). Homologous recombination requires the presence of a homologous partner, which is always present in an obligate diploid. In fact, in *C. albicans*, HR occurs during the mitotic cycle at a rate of 10^−6^ events/cell/generation [4], causing loss of heterozygosity (LOH) in short or long stretches of DNA (through gene conversion or crossover/BIR, respectively), a process that may be clinically relevant [5]. *C. albicans* possesses homologues of the Rad52 epistasis group of HR-related proteins (Rad51, Rad52, Rad54, Rad55, Rad57 and Rad59) [6]. As in *S. cerevisiae*, *RAD52* is involved in most homology-dependent recombination in *C. albicans*, and Ca*rad52* mutants exhibit the most severe phenotypes in recombination, DNA repair, and genetic instability [7]–[9].

The second recombination pathway, NHEJ, can rejoin the two ends of a DSB by simple ligation after little or no nucleolytic processing of the end. In budding yeast, the Yku70/Yku80 complex, Lig4 and its associated Lif1/Nej1 complex, as well as the Mre11/Rad50/Xrs2 complex (MRX complex) are required for NHEJ [10]. Among the homologues of these genes in *C. albicans*, only *LIG4* and *KU80* have been partially characterized [8], [11]. In *S. cerevisiae*, NHEJ is repressed in diploids where the HR pathway is active. This is presumably advantageous because diploid cells contain one intact copy of each gene for the repair of damaged DNA through HR, a high fidelity repair mechanism. Therefore, the retention of NHEJ genes in an obligate diploid yeast such as *C. albicans* suggests that these genes may play important roles in non-NHEJ processes that are important for the biology of the fungus. In fact, besides its role in end-joining, the Ku proteins have been implicated in a variety of functions at telomeres [12]–[14].

Telomeres are nucleoprotein structures located at the ends of chromosomes that are crucial for maintaining chromosome stability. Telomeres in most organisms consist of G-rich repeats that terminate with a single-stranded 3′ overhang [15], [16]. Conventional DNA polymerases cannot fully replicate the very ends of linear DNA molecules. Thus, without a specific compensatory mechanism, the ends of chromosomes shorten during each cell division. Most eukaryotes utilize telomerase, a specialized reverse transcriptase consisting of a catalytic protein subunit (Tert), an RNA template (Ter), and several accessory proteins for telomere addition [13]. Previous studies from yeast to mammals have implicated the Ku complex in performing a multitude of functions at telomeres. In the budding yeast *S. cerevisiae*, the Yku70/Yku80 heterodimer has been shown to protect chromosomal ends from nucleolytic degradation; both *yku70* and *yku80* mutants accumulate high levels of single-stranded telomere DNA (G-tails) due to uncontrolled degradation of the C-strand [17]. In addition, the *S. cerevisiae* Ku complex promotes telomerase recruitment and telomere elongation through an interaction between Yku80 and telomerase RNA; both the *yku70* and *yku80* mutants possess shorter than normal telomeres [14]. Furthermore, binding of Ku to telomeres facilitates the recruitment of Sir3 and Sir4 to the subtelomeric regions to enhance telomeric silencing [18]–[20].

Interestingly, Ku's precise roles in telomere regulation appear to be somewhat divergent evolutionarily [13]. For example, in contrast to *S. cerevisiae*, the disruption of Ku in *Arabidopsis thaliana* results in long and heterogeneous telomeres [21]. Moreover, both the *A. thaliana* and human Ku complex have been shown to suppress the formation of extrachromosomal telomeric circles (t-circles) [22], [23]. T-circles have been observed in a wide range of organisms, often in association with telomere dysfunction, and are believed to arise through telomere recombination [24]. High levels of t-circles are also characteristic of some cancer cells (ALT cells) that lack telomerase [25]. The elevation of t-circles in plant and human *KU* mutants suggest that this complex suppresses the access or activity of recombination factors within the terminal repeats. In contrast to these organisms, a role for the budding yeast Ku complex in suppressing telomere recombination is less clear. In *S. cerevisiae*, the disruption of *YKU* genes alone have not been reported to cause higher telomere recombination, but the same disruption in a *cdc13-1* strain exacerbated aberrant telomere recombination [26]. In *K. lactis*, the loss of Ku80 induces a moderate increase in subtelomeric recombination [27]. In these two fungi, whether the Ku complex plays a role in suppressing recombination within the terminal repeats in wild type cells remains an open question.

In this work, we have characterized the phenotypes of Ku70 mutants of *C. albicans* with respect to growth, DNA damage-sensitivity, and telomere dysfunction. Our findings indicate a relatively minor role, if any, for the Ku complex in DNA damage repair, but revealed complex dosage-dependent effects of the Ku proteins in regulating telomere lengths and structure, including the accumulation G-tails and t-circles. We observed substantial functional distinctions between the *S. cerevisiae* and *C. albicans* Ku proteins with regard to telomere regulation. In addition, intriguing parallels were noted between the phenotypes of the *C. albicans ku70* mutant and comparable mutants from plants and humans.

## Results

### Identification of the *C. albicans KU70* ortholog

**Figure 1:**
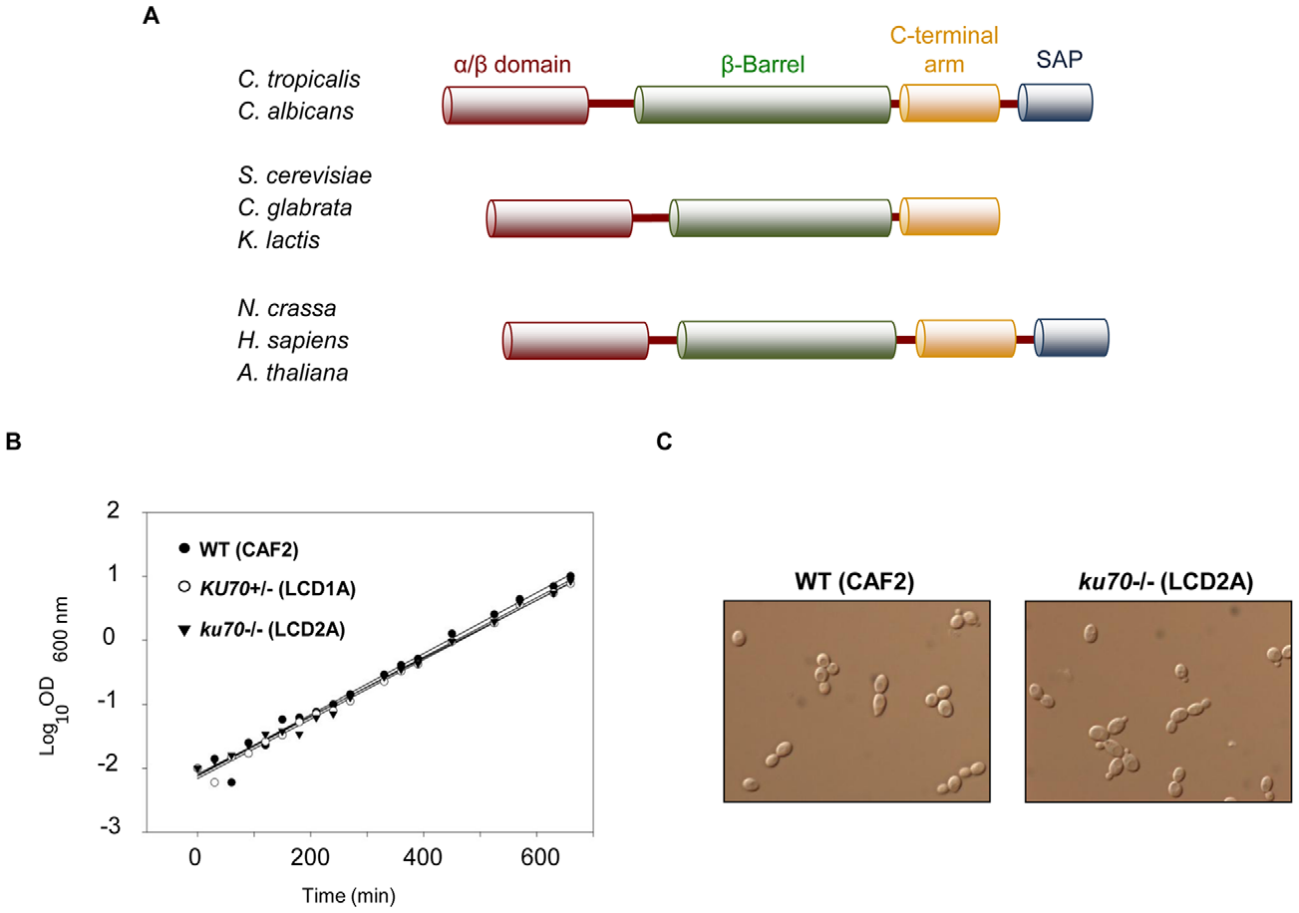
Comparison of the Ku70 proteins and basic phenotypic analysis of *C. albicans ku70* mutants. (**A**) Schematic comparison of the Ku70 proteins from the indicated species showing the relative locations of the conserved motifs. Notably, the SAP domain is present in all *Candida* species except *Candida parapsilosis*, but is absent in all *Saccharomyces* and *Kluyveromyces* yeast. (**B**) Growth curve of *KU70* mutants. The OD_600 nm_ values of the WT (CAF2), *KU70+/−* (LCD1A), and *ku70-/-* (LCD2A) cultures were plotted against time logarithmically. Note that in most figures, we use *KU70+/−* and *ku70-/-* to designate the heterozygous and homozygous null strain, respectively. (**C**) Cell morphology of the parental strain CAF2 and the *ku70-/-* mutant (LCD2A). Both strains were inoculated in liquid YPD medium, incubated at 30°C for 24 hours, and photographed under an optical microscope (x100).

A BLAST search identified an open reading frame (ORF19.1135) in the genome of *C. albicans* strain SC5314 with significant similarity to the *S. cerevisiae* Ku70 protein. This ORF, with 2436 nucleotides, is located on the right arm of chromosome 1 and encodes a predicted protein of 811 amino acids. Clustal analysis revealed that *Ca*Ku70 was 24, 25, 16, 20, 19, and 19% identical to *Candida glabrata* (*Cg*Ku70), *S. cerevisiae* (*Sc*Ku70), *Aspergillus nidulans* (*An*Ku70), *Neurospora crassa* (*Nc*Ku70), *Arabidopsis thaliana* (*At*Ku70) and *Homo sapiens* (*Hs*Ku70) Ku proteins, respectively (Figure 1). Like these homologues, *Ca*Ku70 contains an N-terminal α/β domain, a central β-barrel domain, and a well-conserved C-terminal arm (Figure 1A and Figure S1). The central β-barrel domain of *Ca*Ku70 contains several long insertions relative to other homologues, thus accounting for its larger size. Based on the crystal structure of the human KU70-KU86 complex, these insertions are located near surface loops and are unlikely to disrupt the overall architecture of the proteins (data not shown) [28]. Also present in the C-terminal region of *Ca*Ku70 is the so-called SAP motif, a putative DNA/RNA binding structure found in several nuclear and cytoplasmic proteins. Interestingly, even though the SAP motif is conserved in many Ku70 proteins (e.g., *A. thaliana, H. sapiens*, *A. nidulans* and *N. crassa*), it has apparently been lost in a branch of budding yeast that includes *S. cerevisiae*, *C. glabrata*, and *K. lactis* (Figure 1A and Figure S1) and *Ashbya gossipii* (not shown)[29], which have significantly shorter Ku proteins (576-606 amino acids). A conserved sequence (α helix 5) that is crucial for NHEJ in *S. cerevisiae* is present in the N-terminal region of *Ca*Ku70 as well [30].

*C. albicans* is a diploid organism with a high degree of polymorphism; many genes exhibit allelic differences (http://www.candidagenome.org). As shown in Table 1 (see also Materials and Methods), in strain CAI4, we identified two distinct alleles of *KU70*, referred to as A and B, which exhibit 13 SNPs in the coding region. Of these, eight were synonymous (positions +903, +969, +1041, +1044, +1779, +1827, +1839, and 1869) and five non-synonymous (positions +844, +866, +1697, +1722, and +2264). The latter SNPs result in differences in the protein sequence (Asn282 vs Asp282, Asn289 vs Ile289, Arg566 vs His566, His574 vs Gln574, and Gly755 vs Asp755, respectively). Only the first six SNPs (+844 to +1044) appear in the Assembly 21 of the *C. albicans* Database (strain SC5314) where the non-synonymous SNPs at positions +844 and +866 are designated R and W, respectively. Sequencing of alleles A and B present in the heterozygous strains LCD1A and LCD1B respectively revealed that the new SNPs were due to single base substitutions in allele A. In addition, three new SNPs, due also to changes in allele A, were detected in the terminator region of *KU70*. Therefore, the extra-SNPs detected here may be due to mutations occurring during the construction of strain CAI4. Alternatively, they could have been eliminated by gene conversion in the SC5314 isolate used for sequencing.

**Table 1:** Identification of the SNPs present in the two alleles of the *KU70* gene of strain CAI4.

Relative position within ORF	SNPs		Amino acids	
	Allele A	Allele B	Allele A	Allele B
+844	A	G	Asn	Asp
+866	A	T	Asn	Ile
+903	A	T	Gly	Gly
+969	C	T	Phe	Phe
+1041	G	T	Gly	Gly
+1044	A	G	Gly	Gly
+ 1697	G	A	Arg	His
+ 1722	T	A	His	Gln
+ 1779	T	C	Asn	Asn
+ 1827	G	A	Leu	Leu
+ 1839	G	A	Glu	Glu
+ 1869	T	C	Ile	Ile
+ 2264	G	A	Gly	Asp
+ 2488	G	T		
+ 2512	G	A		
+ 2726	G	A		

The ORF of Ca*KU70* is from positions +1 to +2436. SNPs at positions +2488, +2222, and +2436 are in the terminator region.

### Phenotypic analysis of *ku70* mutants

To initiate a functional analysis of *CaKU70*, we generated a series of heterozygous and homozygous mutants by using a URA-blaster cassette. Several *C. albicans* DNA repair mutants have been shown to exhibit reduced growth rates or abnormal morphology. We therefore first analyzed our *ku70* mutants with respect to these phenotypes [7], [8]. We found that Ca*KU70* is not essential for viability; the generation time of the null homozygous strain (LCD2A, 67 min) was only slightly longer than those of the heterozygote (LCD1A, 65 min) and wild type (CAF-2, 63 min) (Figure 1B). When grown on YPD plates, the null strain exhibited a colony and cellular morphology that is indistinguishable from the wild type. In liquid YPD medium at 30°C, only yeast cells were observed in both parental and null strains (Figure 1C). Thus, the growth behaviors of the *ku70* mutants resemble those of the *ku80* and *lig4* mutants, which, similar to *ku70*, are presumably defective in NHEJ [11], [12]. In contrast, mutants defective in HR (*rad52*, and *rad51*) or in genes common to both HR and NHEJ (*rad50, mre11*) exhibit filamentous morphology and grow as wrinkled colonies under the same conditions [7], [9]. When grown on solid Spider and M-199 plates, *ku70* mutants formed filaments similar to those produced by wild type strains. Finally, in comparison to the wild type strain (CAF2), no difference in filamentation was observed when this morphologic form was induced by addition of serum to YPD liquid cultures (data not shown).

**Figure 2:**
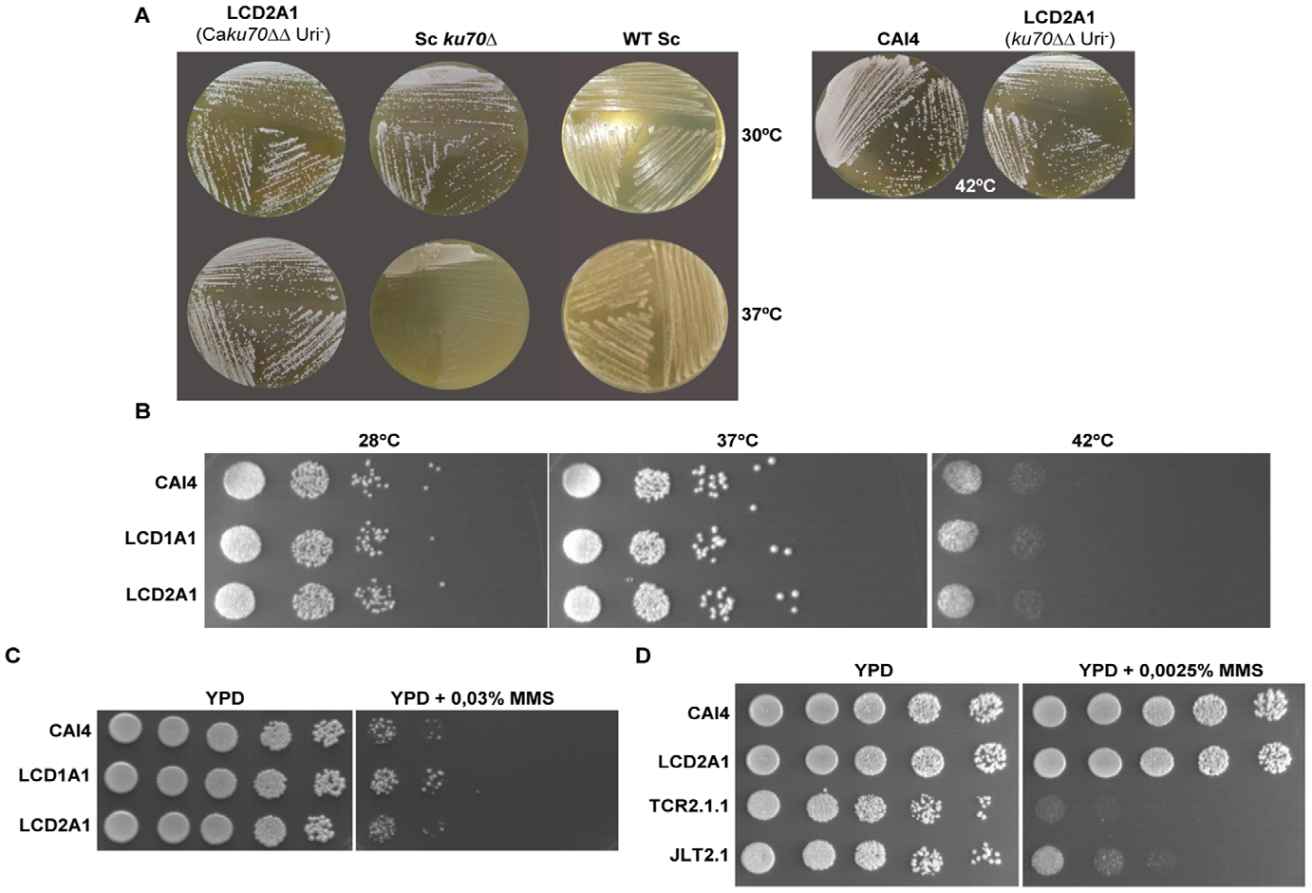
Thermosensitivity of the *C. albicans ku70* mutants. (**A**) *ku70* null mutants of *C. albicans* (LCD2A1) and *S. cerevisae* were grown on YPD plates at 30°C and 37°C for 48 hours. A control of the wild type *S. cerevisiae* is shown. (B) Ten-fold serial dilutions of the indicated strains were applied to YPD plates, and grown 48 h at the indicated temperatures. C) **MMS sensitivity of the** ***ku70*** **heterozygous and null homozygous deletion mutants. D**) **MMS sensitivity of** ***ku70*** **,** ***rad52*** **and** ***ku70 rad52*** **deletion mutants. In C and D,** five-fold serial dilutions of each culture were applied onto YPD plates containing the indicated concentrations of MMS and incubated at 28°C for 72h.

Because the *S. cerevisiae ku70* null mutant is known to be temperature sensitive ([31] and Figure 2A), we analyzed the thermo-sensitivity of our *C. albicans* mutant. At both 37°C and 42°C, both a heterozygous *KU70/ku70::hisG* (LCD1A.1) and a null *ku70::hisG*/*ku70::hisG* mutant (LCD2A.1) behaved like the wild type (CAI4) strain (Figures 2A and 2B). The same was true for the heterozygous and null strains constructed in the BWP17 background (not shown). Thus, the *C. albicans ku70* null mutant is not thermo-sensitive.

We have reported that *rad52* null strains of *C. albicans*, which are defective in HR, are very sensitive to the alkylating agent MMS [7]. As shown in Figure 2C, both heterozygous (LCD1A1) and null *ku70* (LCD2A1) strains were indistinguishable from its parental CAI4 when grown in MMS, even at concentrations as high as 0.03%. A previous analysis indicates that the *Saccahromyces yku70* mutant is sensitive to MMS only in the absence of recombination [32]. We therefore analyzed a *C. albicans ku70 rad52* double mutant (JLT2.1), but found that this mutant did not exhibit greater MMS sensitivity than the *rad52* mutant (TCR2.1.1); both strains showed some growth at 0.0025% MMS (Figure 2D), but were killed by 0.01% MMS (not shown). The *ku70* mutant strains were also treated with 25J/m^2^ of UV radiation. At this dose of irradiation, no difference in viability was detected between the *ku70* mutant and the wild type CAI4 strain. In contrast, all *rad52* mutants (single and double mutants) exhibited increased sensitivity (not shown). Thus, NHEJ is not a major pathway for the repair of MMS or UV-induced lesions.

### Role of *KU70* in telomere length regulation

**Figure 3:**
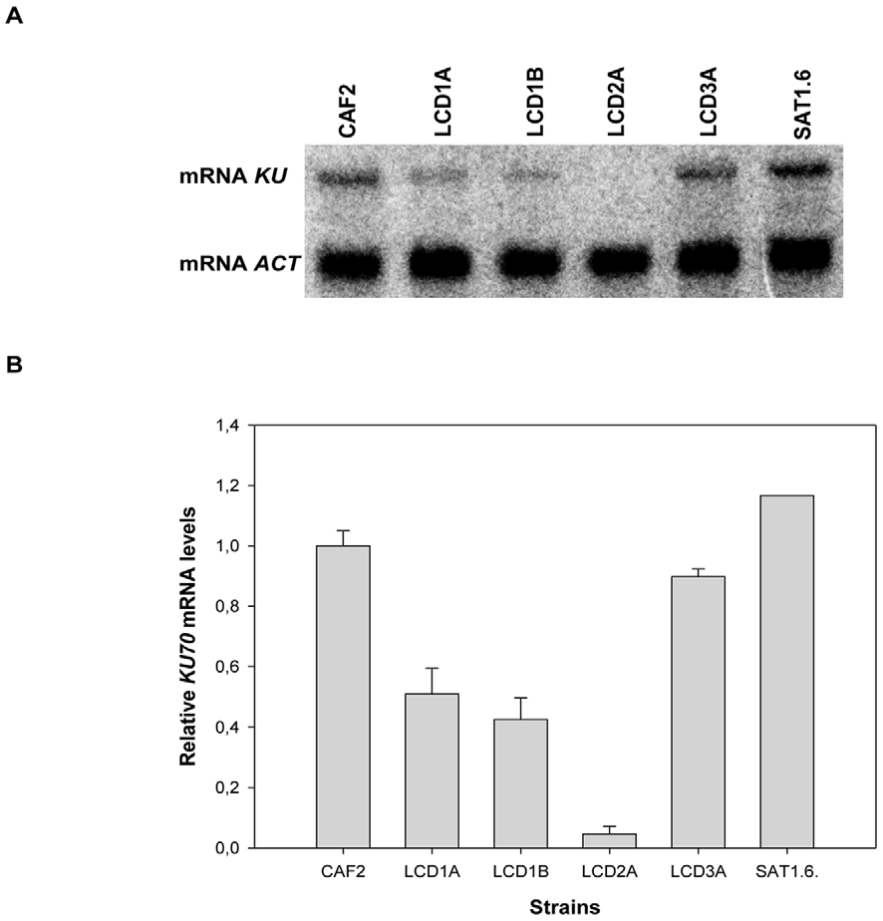
Analysis of the *KU70* transcript levels in the mutant strains. (**A**) A Northern blot analysis of *KU70* transcripts in wild type CAF2 (*KU70*/*KU70*), heterozygotes LCD1A and LCD1B (*KU70*/*ku70*Δ), null mutant LCD2A (*ku70*Δ/*ku70*Δ) and reconstituted strains LCD3A (*KU70*/*ku70::KU70*) and SAT1.6 (*ku70*::*KU70*/*ku70*::*KU70*). The levels of actin (*ACT1*) mRNA were used as a loading control (lower panel). (**B**) The relative *KU70* transcript levels were calculated (Material and Methods) and plotted.

Because the SNP differences between the *KU70* alleles, we generated a collection of heterozygous (LCD1A and LCD1B, each with a different allele) and reconstituted strains (see below) and analyzed the transcript levels of *KU70* as well as the telomere phenotypes of these strains. The level of the *KU70* mRNA was decreased by one half in the heterozygous strains LCD1A (retains allele A) and LCD1B (retains allele B), whereas no message was detected in the null strain (Figure 3). A reconstituted strain LCD3A, constructed by reintroducing allele B into LCD1A (i.e., it carries both alleles of *KU70*), regained the wild type expression level, whereas its counterpart LCD3A.1, constructed by reintroducing allele B into the null strain LCD2A1 (i.e., it carries a single copy of allele B), exhibited a level similar to the heterozygous strain LCD1B (allele B) (not shown). Reintroduction of a second copy of allele B into LCD3A.1 (strain SAT1.6) increased the amount of the *KU70* message to wild type levels. Therefore, the amount of the *KU70* transcript is roughly proportional to the gene dosage.

**Figure 4:**
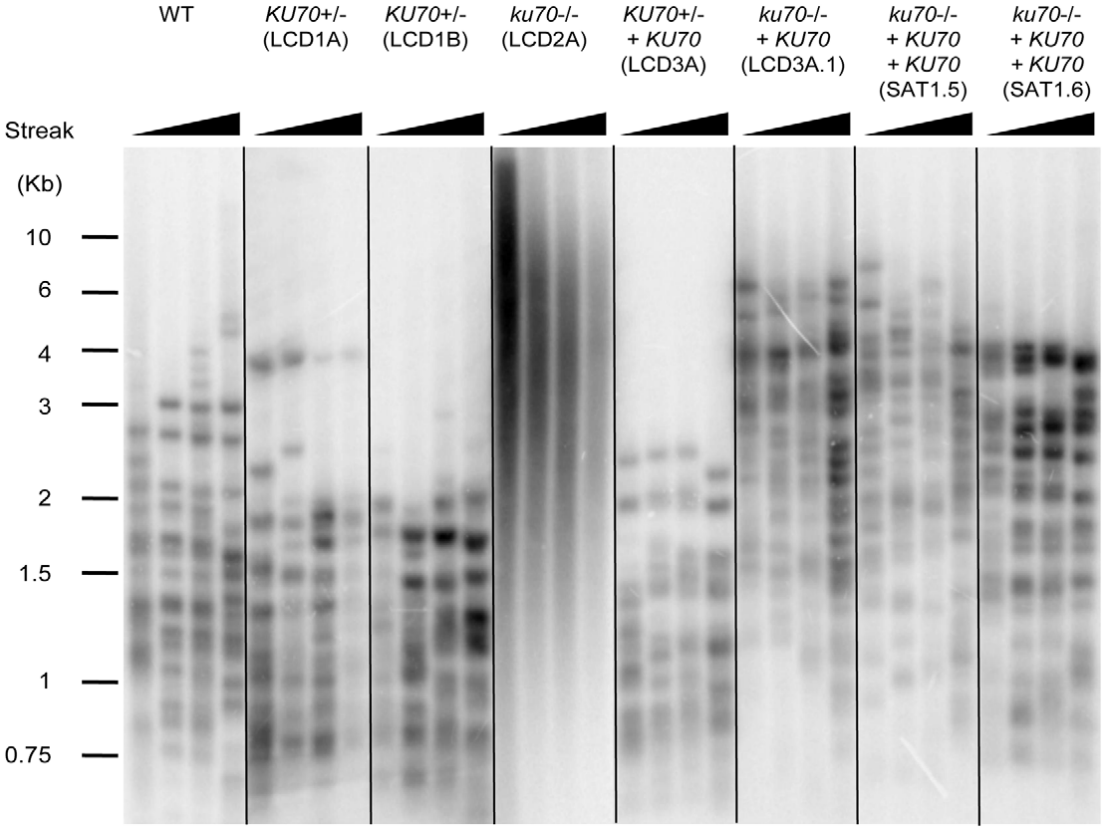
Analysis of telomere lengths in the *ku70* mutants and reconstituted strains. The indicated strains (all Uri^+^ and all derived from CAI4) were passaged on YPD plates by successively streaking for single colonies. Chromosomal DNA samples were prepared from the strains following 2, 4, 6 and 8 streaks, and subjected to telomere Southern analysis as described [34].

To analyze the effect of *C. albicans KU70* deletion on the length and distribution of telomeres, we compared the wild type, the heterozygous and the null homozygous strains after repeated passages (Figure 4). All of the strains analyzed in this experiment carry the *URA3* gene (Uri^+^). The heterozygous strains containing either allele of *KU70* (LCD1A and LCD1B) maintained on average slightly shorter telomeres than the parental strain (CAF2). In contrast, the *ku70* null strain contained telomeres that are much longer than the parental strain (Figure 4). The distribution of telomere fragments also differs between the various strains. Specifically, distinct clusters of telomere bands can be discerned in the wild type and heterozygous, but not in the null strain, indicating that the latter strain has increased telomere length heterogeneity (Figure 4). To avoid the potential effects of the position in the genome of the marker gene (*URA3*), we also compared the Uri^−^ derivatives of the Ku mutants to the parental strain CAI4, as well as strains generated from BWP17 (another reference strain), and found similar telomere phenotypes for the heterozygous and null mutants (data not shown). Several features of the telomere phenotypes are worthy of further comments. First, the shortening of telomeres in the heterozygous mutants were apparent in the earliest passage (∼75–100 generations after the derivation of the mutant) and did not change appreciably afterwards, suggesting rapid kinetics that is distinct from other telomere mutants such as telomerase-null strains. However, because telomere shortening in the *KU70*+/*−* heterozygous strain is very modest, it may take a relatively small number of generations to reach to the new equilibrium distribution even if the rate of shortening is as low as in other mutants. In addition, because of the modest length difference between the telomeres of the wild type and heterozygous strains, more studies will be necessary to confirm this defect definitively. Finally, the long and heterogeneous telomeres of the homozygous knockout strain suggest the possible existence of aberrant telomere DNAs that do not migrate into the gel during electrophoresis (found e.g., in the *rap1-/-* mutant, which also has long and heterogeneous telomeres [33]). However, Southern analysis using blots that included the wells of the gel did not reveal such aberrant telomeric DNA in the *ku70-/-* mutant (Figure S2).

Next, we used in-gel hybridization to analyze the levels of G-strand overhangs in the various strains. Interestingly, we observed an increase in the amount of G-strand overhangs in the *ku70* heterozygotes, but not in null strain relative to the parental CAI4 (Figure S3). The reason for this somewhat paradoxical finding is not understood, but similar results were reported for human *KU86* heterozygous and homozygous null mutants [34], [35]. The accumulation of G-strand overhangs in the heterozygous strain is reminiscent of *C. albicans* telomerase mutants as well as *S. cerevisiae yku* mutants, and consistent with a role for the *C. albicans* Ku complex in protecting telomeres against C-strand degradation [36], [17]. Altogether, our findings suggest that *C. albicans KU70* regulates telomere length and structure in a dosage-dependent manner.

We then analyzed the consequences of re-integrating the *KU70* gene back into the heterozygotes and null strains. Surprisingly, the telomere defect of the heterozygote was not suppressed by reintroducing a missing allele, as shown for strain LCD3A (Figure 4). Similarly, reintroducing one copy of *KU70* (allele B) into the null strain LCD2A did not completely restore the telomere phenotype of the heterozygote. Although the telomeres of the resulting strain LCD3A.1 showed reduced heterogeneity, they remained elongated through many passages (Figure 4 and Figure S4). Introducing a second copy of *KU70* into LCD3A.1 caused a progressive decline of telomere length followed by stabilization (SAT1.5 and SAT1.6 in Figure 4, see also Figure S4). These complex effects of *KU70* reintroduction reinforce the notion that the regulation of telomere length by *KU70* is dosage dependent. They also suggest that the initial telomere lengths of the strain influence the final equilibrium length reached after alterations in *KU70* dosage.

### The mechanisms of telomere elongation in the *ku70* null strain

**Figure 5:**
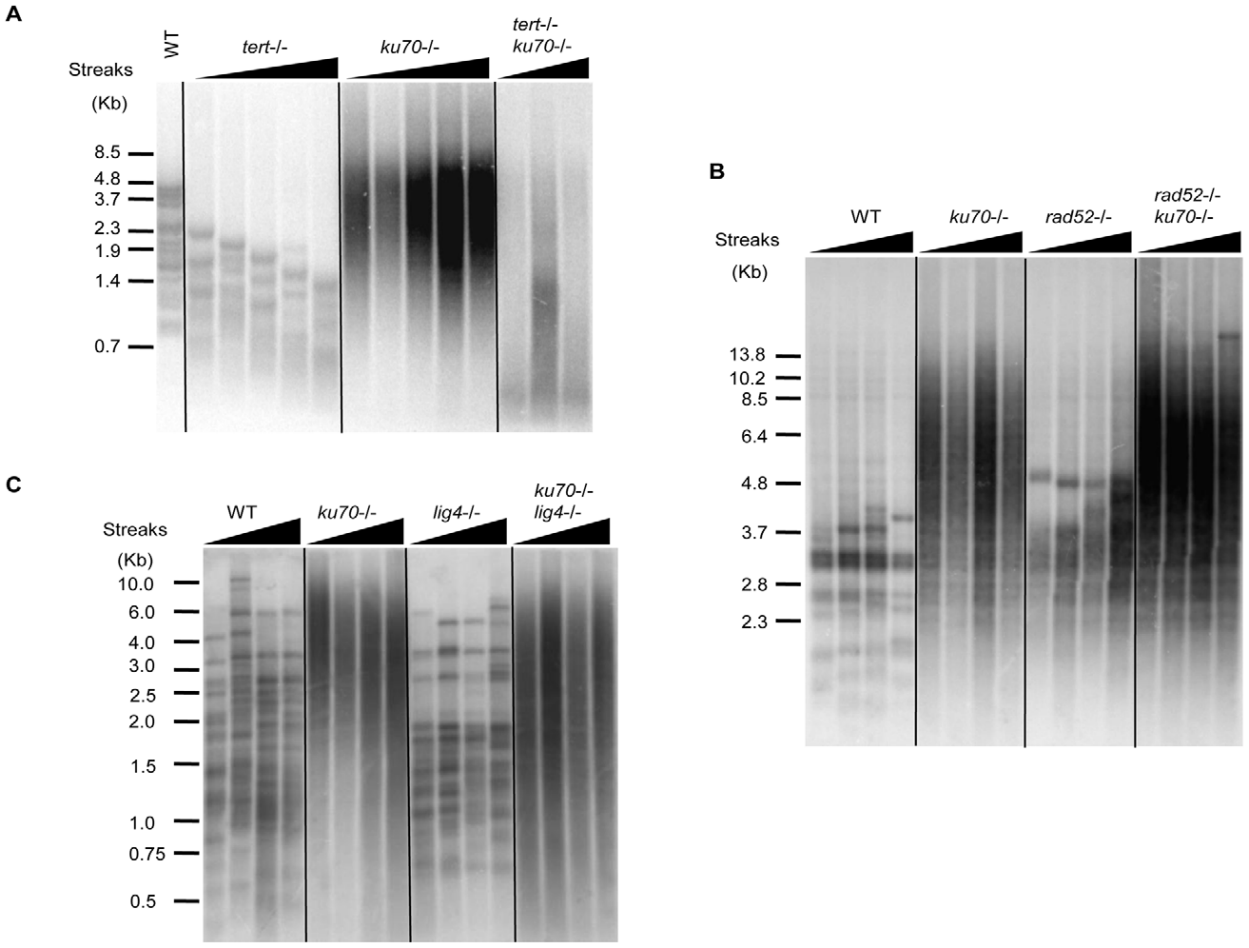
Comparative analysis of telomere lengths in the *ku70* and other related mutants. (**A**) The indicated strains were passaged on YPD plates by successively streaking for single colonies. Chromosomal DNA samples were collected from different passages and subjected to telomere Southern analysis as described. The streaks from which the DNA samples were prepared are as follows: *tert-/-*, streak 5, 7, 9, 11 and 13; *ku70-/-* (LCF2.1) streak 2, 4 6, 8 and 10; *tert-/- ku70-/-* (LNL2.1), streak 2, 4 and 6. All strains are derivatives of the BWP17 parental strain. (**B**) Same as A except that all DNA samples were prepared following 2, 4 6, and 8 streaks on YPD plates, and all strains are derivatives of the CAI4 parental strain, as follows: *ku70-/-* (LCD2A.1); *rad52-/-* (TCR2.1.1); *rad52-/- ku70-/-* (JLT2.1). (**C**) Same as A except that all DNA samples were prepared following 1, 5, 9 and 13 streaks, and all strains are derivatives of the CAI4 parental strain, as follows: *ku70-/-* (LCD2A.1); *lig4-/-* (CEA2.5); *ku70-/- lig4-/-* (MLE2.1).

To determine the mechanisms of telomere elongation in the *ku70* mutant, we created various combination mutants and compared their telomeres to the *ku70* single mutant. As shown in Figure 5A, telomeres of the *ku70 tert* combination mutant are similar in size to the *tert* mutant and substantially smaller than the *ku70* single mutant, indicating that the lengthening is telomerase-dependent. However, in terms of size heterogeneity, the *ku70 tert* combination mutant resembles more closely the *ku70* mutant, suggesting that telomerase is not responsible for generating telomere size heterogeneity in the combination mutant. Thus, at least two mechanisms are acting to create the characteristic telomeres of the *ku70* mutant. Interestingly, whereas neither the *ku70* nor the *tert* single mutant displayed growth defect at 40°C, the growth of a *ku70 tert* double mutant was significantly affected at this temperature. Thus, unlike the *S. cerevisiae* mutant, the thermo-sensitivity of the *C. albicans ku70* mutants was only observed in the absence of telomerase (Figure S5). It has been shown earlier that the *ts* phenotype of the *S. cerevisiae yku* mutants is caused by short telomeres [37]. Hence the difference in temperature sensitivity between the *S. cerevisiae* and *C. albicans ku* mutants can be explained by the fact that the *C. albicans ku70-/-* strains have long rather than short telomeres.

We have shown earlier that the absence of Rad52 also resulted in an increase in the telomere lengths [7]. As shown in Figure 5B, this effect, although reproducible, was rather modest as compared with that caused by the absence of Ku70. A double mutant *ku70 rad52* has telomeres that resemble the *ku70* null strain, suggesting that the *ku70* is epistatic to *rad52* with regard to telomere lengthening and the induction of telomere length heterogeneity.

We next asked whether the effect caused by the absence of Ku70 was paralleled by the absence of Lig4, another component of the NHEJ pathway. Telomere fragments of the *lig4* mutant were similar in length to those of the parental strain, although they appeared slightly sharper. Deleting the *LIG4* gene in the *ku70* background did not modify the length or the heterogeneity of the telomeres (Figure 5C). Therefore, the lengthening and the increased heterogeneity of *ku70* telomeres appear unrelated to NHEJ.

### Detection of circular telomeric DNA in the *ku70* null mutant

**Figure 6:**
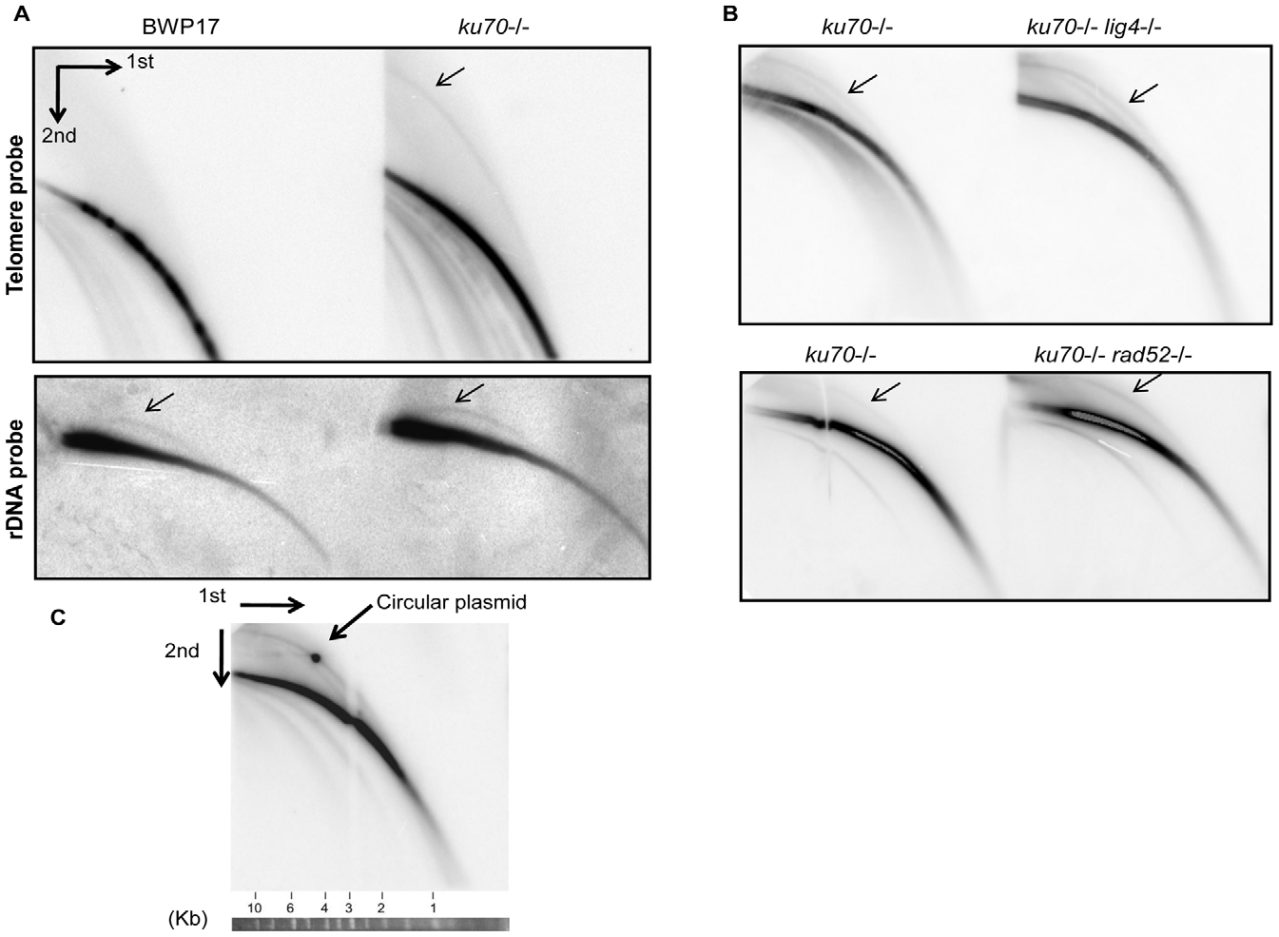
Identification of t-circles in the *ku70* null mutants. (**A**) (**Top**) Genomic DNA samples were prepared from the parental wild type strain (BWP17) and its *ku70-/-* derivative (LCF2.1), digested with *Alu*I and *Nla*III, and then subjected to 2-D electrophoresis followed by Southern blotting to identify linear and circular telomeric DNA. (**Bottom**) The sample DNA samples were subjected to 2-D gel electrophoresis (without prior digestion) and analyzed using an rDNA probe. (**B**) Genomic DNA samples from the indicated strains were subjected to 2-D gel electrophoresis followed by Southern blotting to assess the levels of linear and circular telomeric DNA. All mutant strains were derived from the CAI4 parental strain, as follows: *ku70-/-* (LCD2A.1); *ku70-/- lig4-/-* (CEA2.5); *ku70-/- rad52-/-* (JLT2.1). (**C**) Genomic DNAs from the *ku70-/-* strain (LCD2A.1) were digested and analyzed by 2-D gel electrophoresis together with a nicked 4.4 kb circular plasmid (pGEM-URA3, isolated by gel electrophoresis). Radioactive probes for the plasmid and for telomere DNAs were both included in the hybridization mixture. A strip of DNA size standards corresponding to the first round of electrophoresis is shown at the bottom.

The finding that the absence of Ku70 caused an increase in both the heterogeneity and length of telomeres in *C. albicans*, although contrary to the results obtained in *S. cerevisiae*, mimics the situation reported for *A. thaliana*, where loss of this protein also caused telomeres to be 2 to 3-fold longer and much more heterogeneous than the wild type cells [22]. In *A. thaliana*, Ku70 suppresses ALT (Alternative Lengthening of Telomeres), an HR-based mechanism that results in the formation of telomeric circles (t-circles). To determine if this mechanism operates also in *C. albicans*, we analyzed the structural properties of telomeric DNA in the *ku70* null strain. For this purpose, *Alu*I and *Nla*III-digested genomic DNA was resolved using two-dimensional (2D) gel electrophoresis and then the telomeric DNA detected using a specific probe. As shown in Figure 6A, the parental strain BWP17 showed arcs corresponding only to linear DNA whereas its isogenic *ku70* null strain showed arcs corresponding to both circular DNA and linear DNA. By contrast, the level of ribosomal circles was unaffected by *KU70* deletion [Figure 6A]. Thus, similar to *A. thaliana*, the *C. albicans* Ku70 plays an important role in specifically suppressing the formation of t-circles.

As noted earlier, the absence of Lig4 in the *ku70* background did not alter the length or the heterogeneity of telomeres. 2D analysis revealed similar or slightly elevated levels of t-circles in the *lig4 ku70* double mutant in comparison to the *ku70* single mutant (Figure 6B). This phenotype is stable for at least ∼150 generations (six passages). Thus, Lig4 does not influence significantly t-circle formation in the *ku70* mutant.

The formation of t-circles in the *ku70* mutants is presumably mediated through a recombination-based mechanism. Because, like other budding yeast, Rad52 is apparently the most important gene for HR in *C. albicans* [7], we analyzed the level of t-circles in the *ku70 rad52* double mutant. Interestingly, the abundance of t-circles in this double mutant is again similar or slightly higher than that of the *ku70* single mutant, indicating that Rad52 is dispensable for t-circle maintenance (Figure 6B).

Other non-linear DNAs such as branched molecules are also known to migrate slower in the second dimension in the 2D gel system. To confirm our designation of t-circles, we analyzed the *ku70-/-* DNA sample together with a nicked circular DNA plasmid (Figure 6C). The signal for the nicked circular plasmid indeed localized to the putative circular arc, supporting our assignment.

## Discussion

Although *Ca*Ku70 exhibited a low level of sequence identity/similarity to other homologues, it retains the shared motifs and most likely the same structural organization as other Ku70 proteins. In the present study, we have characterized the potential roles of *Ca*Ku70 based on previous findings in other organisms. In contrast to *S. cerevisiae ku70* mutants, *C. albicans ku70* was not thermosensitive. Furthermore, Ku70 does not play any significant role in the repair of lesions caused by MMS or UV light in *C. albicans*. However, our analyses did reveal complex regulation of telomere length and structure by *Ca*Ku70.

### The *C. albicans* Ku complex acts in both telomerase regulation and telomere protection

Studies in various organisms have revealed disparate impacts of *KU* gene mutations on telomere lengths. In baker's yeast, as well as in organisms ranging from fission yeast, humans to trypanosomes, deletion of *KU* genes results in telomere shortening [14], [35], [38], [39]. In contrast, Ku deficient *Arabidopsis* cells exhibit very long and heterogeneous telomeres [21]. These findings suggest that depending on the experimental system, Ku can play either a positive or negative regulatory role in telomere length regulation. Remarkably, we observed complex effects of *KU70* gene dosage on *C. albicans* telomere length, indicating that the *C. albicans* protein may simultaneously play both a positive and negative regulatory role. Disruption of one copy of *KU70* resulted in telomeres that were slightly shorter than those of wild type cells and this effect was both reproducible and independent of the allele present in the heterozygotes. This observation is reminiscent of the short but stable telomeres of haploid *S. cerevisiae* y*ku70 and yku80* mutants upon prolonged passage [40], [41]. The positive regulatory function of Yku70 and Yku80 involves a direct interaction between Yku80 and telomerase RNA [14]. However, we have not been able to detect a comparable interaction between *Ca*Ku70 or *Ca*Ku80 and *C. albicans* telomerase RNA (data not shown). Further studies will be necessary to determine the molecular basis of the telomere elongation function of *Ca*Ku70 suggested by the phenotype of the heterozygous mutant. In stark contrast to the heterozygous strains, homozygous *C. albicans ku70* deletion strains exhibit long and heterogeneous telomeres resembling those of the corresponding *Arabidopsis ku70* mutants. Such a phenotype has been attributed to loss of telomere capping, leading to aberrant access of telomerase at telomere ends [42]. Consistent with this idea, the telomere elongation in the *C. albicans ku70* mutant appears to be mostly telomerase dependent.

In addition to allowing uncontrolled telomerase action at telomeres, the *C. albicans ku70* mutant resembles the corresponding *Arabidopsis Ku70* and human *ku86* mutants in possessing high levels of t-circles [22], [23]. These t-circles are thought to derive from aberrant recombination, and are hallmarks of the ALT pathway described in human cancers. The induction of both telomerase and recombination activities at *ku70* telomeres suggests that the Ku complex may directly regulate both pathways by facilitating the formation of a protective structure. However, a recent study of human cells indicates that t-circle levels can be elevated by telomerase overexpression, suggesting that long telomeres by themselves are sufficient to induce telomere recombination [43]. Hence the elevated telomere recombination of the *C. albicans ku70-/-* mutant could be a secondary consequence of uncontrolled telomerase activity. In any case, it is clear that loss of Ku70 leads to phenotypes that are quite reminiscent of those caused by the loss of other telomere binding proteins such as Rap1, Stn1 and Ten1 [33], [44].

More findings in support of a protective function for Ku at telomeres came from analysis of single-stranded G-tails. Previous studies indicate that deprotected telomeres are often subjected to preferential degradation of the C-strand, leading to accumulation of G-tails. Remarkably, we found that G-tail accumulation in *C. albicans KU70* mutants was also dosage-dependent, with the heterozygous mutant manifesting high levels of G-tails and the homozygous null mutant exhibiting no evident accumulation. These observations, while paradoxical, are actually identical to those described for the human Ku86 mutants [23], [34]. One speculative explanation is that longer G-tails are formed in the homozygous null mutant, but are more quickly processed by recombination or C-strand fill-in synthesis due to greater accessibility of the tails in the null mutant. More studies will be necessarily to address this possibility.

Notably, the distinctive telomere phenotypes of the *ku70* mutant in *S. cerevisiae* and *C. albicans* indicate that even between closely related budding yeast, the telomere maintenance factors have underwent significant evolutionary divergence and mediate slightly different functions. The *S. cerevisiae* telomere complex has been studied extensively and utilized as a model system for understanding human telomeres. However, the *C. albicans* Ku complex appears to exhibit more functional similarities to the human complex from the standpoint of suppressing aberrant recombination and suppressing G-tail formation. Further analysis of this genetically tractable diploid fungus may thus provide insights on Ku mechanisms that could not be obtained from *S. cerevisiae*.

### T-circles and telomere recombination in the *ku70* mutant

The phenotypes of *ku70 lig4* and *ku70 rad52* combination mutants provide further insights on the function of *KU70* and raise interesting questions concerning the mechanisms of t-circle formation and maintenance. First, neither *LIG4* nor *RAD52* deletion significantly altered the lengths of telomeres in the *ku70* mutant, consistent with telomerase (rather than NHEJ or HR) being largely responsible for the observed elongation. Additionally, the level of the t-circles observed in the *ku70* mutant was not modified by the subsequent deletion of *LIG4* or *RAD52*. Our failure to eliminate t-circles in the *rad52 ku70* combination mutant is surprising at first glance, since t-circles formation is thought to be mediated by an aberrant, HR-related mechanism [25] and *RAD52* is important for most HR pathways in both *S. cerevisiae* and *C. albicans*. However, it is possible (though unlikely) that t-circles may replicate autonomously in *C. albicans*. Because our combination mutant was derived from sequential disruption of *KU70* and *RAD52*, our results can be explained by the proposition that *RAD52* is required for the generation but not the maintenance of t-circles. In addition, many putative *Arabidopsis* recombination genes, including *MRE11* and *RAD51* paralogs, have been shown to be dispensable for t-circle accumulation [22]. Finally, a *RAD52*-independent recombination pathway for telomere maintenance named ILT was recently uncovered in *S. cerevisiae*, again underscoring the multiplicity of recombination mechanisms and their disparate requirements [45].

In summary, we have shown that *Ca*Ku70 regulates *C. albicans* telomeres through multiple mechanisms, some of which are quite distinct from those of the Yku proteins in *S. cerevisiae*. Continued exploration of this alternative model system should lead to broader insights on the mechanisms and evolution of the Ku complex in budding yeast and other organisms.

## Materials and Methods

### Strains and media

The *C. albicans* strains used in this study are listed in Table S1. *C. albicans* cells were grown routinely at 30°C in YPD (1% yeast extract, 2% Bacto peptone, 2% glucose) or SC (0.67% yeast nitrogen base, 2% glucose) missing or containing uridine (25 µg/ml), histidine (90 µg/ml), arginine (89 µg/ml). Uri^+^ cells were selected in SC medium lacking uridine and Uri^−^ auxotrophs on SC plates containing 0.1% 5-FOA and 25 µg/ml uridine. Nourseothricin-resistant (Nou^R^) transformants were selected on YPD agar plates supplemented with 200 µg/ml of nourseothricin (Werner Bioagents, Jena, Germany) [11], [46], [47]. For testing hyphal formation in *C. albicans,* the cells were grown in liquid YPD containing 10% pre-heated bovine serum at 37°C or on solid M-199 (3.57% of 150 mM Hepes, 0.98% M-199, 2% agar, pH 7–7.2) and Spider (1% mannitol, 1% nutrient broth, 0.2% K_2_HPO_4_, 1.36% agar, pH 7) media.

### Cloning of *KU70*

ORF19.1135, the candidate for *CaKU70* (see Results) was amplified by PCR of genomic DNA isolated from CAI4. Primer pairs for PCR (with restriction sites for *Sac*I and *Hind*III, respectively) were complementary to positions −416 to −395 and +2863 to +2882 in relation to the first nucleotide of the ORF (Table S2). The products were purified, inserted in between the *Sac*I and *Hind*III site of pGEM7Zf(+), and then transformed into *E. coli*.

Several independent transformants were cultured for DNA isolation. Southern blot hybridization of electrophoretic karyotype blots [9] of strain CAI4 with an internal fragment of *KU70* (HDF-A2F and HDF-B3R primers, Table S2) confirmed its location on chromosome 1 (not shown).

### Disruption of *KU70*

We used the *C. albicans* strains CAI4, BWP17, CEA2.5 and a *tert* null mutant for the sequential disruption of this ORF by the ‘URA-blaster’ method [48]. Briefly, a PCR fragment containing 416 bp of the noncoding upstream sequence and 233 bp 5′ ORF (amplified using the AF and AR primer, Table S2) was inserted 5′ to the URA-blaster cassette, and a PCR fragment containing 244 bp of 3′ ORF and 445 bp of downstream sequence (amplified using the BF and BR primer, Table S2) was inserted 3′ to the URA-blaster cassette. As a consequence, the disruption cassette was expected to replace 1959 bp of ORF19.1135.

*C. albicans* transformations were carried out as described [49]. Uri^+^ transformants were first isolated on SC-uridine and then tested for the correct integration of the URA-blaster cassette by Southern analysis. Uri^−^ clones were selected by their ability to grow on 5-FOA plates and used for the disruption of the second allele with the same cassette. The loss of both alleles was confirmed by Southern analysis using *Hind*III-digested genomic DNA and the *Hind*III-*Sac*I fragment of the disruption cassette as the probe. After disruption of the second allele, no wild type allele was detectable in any of the strains. Several independent heterozygous and null homozygous strains were constructed. Because we have used *URA3* as a recyclable marker, and differences in *URA3* expression may influence some phenotypes, in most experiments we compared Uri^−^ mutants and the Uri^−^ control (CAI4). Other experiments were performed using Uri^+^ mutants and the Uri^+^ parental strain (CAF2).

### Reintegration of *KU70* at its native locus in the *ku70* mutants

In order to reintegrate a cloned allele of *KU70* into the heterozygote and null mutant, these mutants were transformed with a cassette containing a copy of cloned *KU70* gene and the *URA3-hisG*. Thus, this ORF was directed to its own locus by the homology between the cassette and the disrupted *ku70*Δ::*hisG* allele. The transformation was carried out as described previously [49] and Uri^+^ transformants were selected on SC-uridine plates. To screen for the desired re-integrants, genomic DNA was digested by *BamH*I and analyzed by Southern blots using a *Hind*III-*Sac*I fragment of the cloned *C. albicans KU70* as the probe. Reconstituted strains derived from a heterozygote mutant contain two copies of ORF 19.1135 and reconstituted strains derived from a null mutant contain only one copy of the ORF. All the reconstituted strains are prototrophic for uridine.

To construct a *KU70/KU70* strain from the null mutant, the strains with one re-integrated *KU70* gene were transformed with a cassette containing a copy of *KU70*, a *SAT1* marker and a 3′ fragment from *KU70* [47]. Nourseothricin-resistant (Nou^R^) transformants were selected on YPD plates with 200 µg/ml of nourseothricin, and correct re-integrants were identified as described previously.

The *KU70* genes of the heterozygous strain LCD1A and all the reintegrants were sequenced to verify that no mutations in the *KU70* ORF had been introduced during the genetic manipulations.

### Characterization of polymorphism in *KU70*

The *KU70*-containing plasmid was sequenced using eight pairs of oligonucleotides (eight forward and eight reverse) (Table S2). These primers are evenly spaced along the ORF to yield reliable and overlapping sequences that allow the assembly of the entire ORF. The data from the sequencing reactions were analyzed using the SeqMan software (DNAStar, *Lasergene*), and the consensus sequence from our clone was compared with the database sequence using the MegAlign software (DNAStar, *Lasergene*). Several potential SNPs were identified. In order to confirm the SNPs, genomic DNA of CAI4 was again amplified by PCR to obtain fragments encompassing residue +782 to +1260 of the ORF. The fragments were sequenced directly. In addition, sequencing of the heterozygous strain LCD1A and *KU70*-reintegrant LCD3A (see Results) indicated the presence of additional SNPs, whose presence was traced to strain CAI4.

### DNA extraction and analysis

Chromosomal DNA was isolated from protoplasts obtained by incubation of cells with Zymolyase [11] or by the ‘Smash and Grab’ method [50]. For *Southern* analysis, genomic DNA was digested with restriction enzymes, subjected to electrophoresis into an agarose gel, transferred to nitrocellulose or nylon membranes and probed with the indicated DNA fragments (labeled with ^32^P using the Random Primed DNA Labeling Kit (Roche). The membrane was mostly analyzed using a Molecular Imager FX (Bio-Rad). Alternatively, an X-Omat X-AR film (Kodak) was used to visualize the bands.

### RNA extraction and Northern analysis

Total RNA was isolated by extraction with hot acidic phenol from 50 ml of *C. albicans* cultures in the exponential phase of growth (OD_600 nm_ = 0.5 approximately). ORF19.1135 expression levels in the *ku70* mutants were measured by Northern using a ^32^P labeled internal fragment of *KU70* gene (HDF-A2F and HDF-B3R primers, Table S2) as the probe. The membrane was analyzed as described for Southern blots and the bands were quantified using the Corel Draw Graphics Suite X3 software.

### MMS and UV light treatment

Cultures of the various strains were grown at 30°C until OD_600 nm_ = 1. For MMS-sensitivity analysis, 5 µl aliquots of the original suspension and of 5-fold serial dilutions of each mutant were applied onto YPD plates containing varying percentages of MMS. For UV-sensitivity analysis, the cell suspensions and serial dilutions were applied to YPD plates, and the plates were irradiated with 25 J m^−2^ of UV light. Both the MMS-containing and UV-irradiated plates were incubated between 48 and 72 hours at 30°C and then photographed.

### Analysis of telomere length and structure

For analysis of telomere lengths over multiple generations, cells were streaked on YPD plates, grown for 2 days at 30°C, and one colony was picked for further re-streaking. Following the desired number of passages, a colony was inoculated into 20 ml YPD, and the culture grown at 30°C for 24 hours. Genomic DNA was isolated as described previously and then digested with a combination of *Alu*I and *Nla*III [51]. Restriction fragments were subjected to electrophoresis into a 0.8% agarose gel and transferred to a nylon filter. Telomeric DNA was detected by hybridization at 50°C to a 5′end-labeled oligonucleotide containing two copies of the *C. albicans* telomere repeat (Table S2) [52].

Single-stranded telomere overhangs were analyzed by in-gel hybridization using a previously described protocol [36].

T-circles were detected using 2D gels as described by Brewer and Fangman with modifications by Cohen and Lavi (1996) [53]. *Alu*I- and *Nla*III-digested or undigested genomic DNA was first separated according to size in a 0.5% agarose gel at 1 V cm^−1^ for 22 hours. Strips of the gel containing DNAs that range in size from about 250 bp to slightly larger than 10 kb were excised, and then subjected to electrophoresis in the orthogonal direction in a 1.2% agarose gel containing 0.3 µg/ml ethidium bromide at 6 V cm^−1^ for 4 hours. Circular DNA molecules are expected to migrate slower during the second round of electrophoresis, forming a separate arc from linear molecules. The DNA was transferred onto a nylon filter and hybridized with *Candida* telomere or rDNA probes (Table S2). The hybridization condition was the same as previously describe for telomere Southern blots.

## Supporting Information

Figure S1 **Multiple sequence alignment of Ku70 proteins from** ***C. albicans*** **,** ***C. tropicalis*** **,** ***S. cerevisiae*** **,** ***C. glabrata*** **,** ***K. lactis*** **,** ***A. nidulans*** **,** ***N. crassa*** **,** ***A. thaliana*** **and** ***H. sapiens*** **; the α/β domain, β-barrel, C-terminal arm and SAP domains are highlighted in different colors.**
(TIF)

Figure S2 **Analysis of telomere lengths in various Ku70 mutants.** Duplicate Genomic DNA samples were prepared from the indicated strains following 2 streaks on YPD plates, and subjected to telomere Southern analysis. The entire gel (including the wells) was subjected to transfer and hybridization. The location of the well in the PhosphorImager scan is indicated by an arrow.
(TIF)

Figure S3 **Analysis of G-strand overhangs in the** ***ku70*** **heterozygous and homozygous mutants.** **(A)** Genomic DNA samples from cultures of the indicated strains were prepared from different passages, and subjected to in-gel hybridization analysis of telomeric G-strand overhangs as described (36). The *KU70+/−* samples were prepared following 2, 4, 6 and 10 streaks, whereas the *ku70-/-* samples were prepared following, 2, 4, 6, 8 and 10 streaks on plates. Both the *KU70+/−* strain (LCD1A.1) and the *ku70-/-* strain (LCD2A.1) are derived from the CA4 parental strain. **(B)** After the detection of G-strand overhangs, the DNA fragments in the gel were denatured and hybridized again with the same probe to identify all terminal restriction fragments.
(TIF)

Figure S4 **Analysis of the stability of telomere lengths in reconstituted strains.** Genomic DNA samples were prepared from the indicated strains following 2, 4, 6, 8, 10 and 12 streaks on YPD plates, and subjected to telomere Southern analysis.
(TIF)

Figure S5 **Analysis of the thermosensitivity of telomerase and** ***ku*** **mutants.** The indicated strains were tested for thermosensitivity by growing five-fold serial dilutions of each culture on YPD plates at the indicated temperatures.
(TIF)

Table S1 ***C. albicans*** **,** ***S. cerevisiae*** **, and** ***E. coli*** **strains used in this study.**
(DOCX)

Table S2 **Oligonucleotides used in this study.**
(DOCX)
