# Supplementary figures and images for: The Candida albicans Ku70 Modulates Telomere Length and Structure by Regulating Both Telomerase and Recombination
Source: PLoS One. 2011 Aug 23;6(8):e23732. doi: 10.1371/journal.pone.0023732 (PMC3160324; doi:10.1371/journal.pone.0023732)

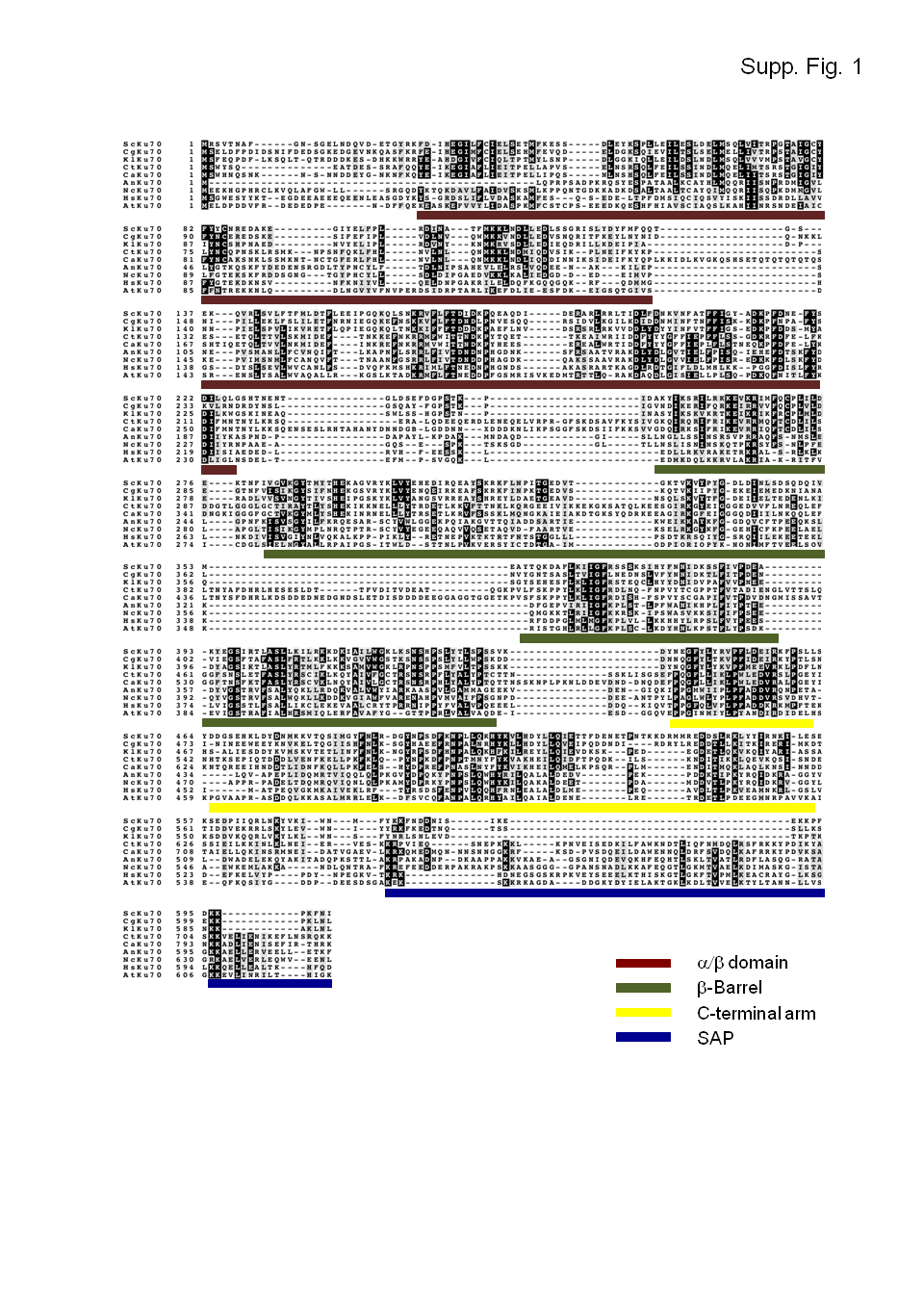

Supplement: Figure S1 — Multiple sequence alignment of Ku70 proteins from C. albicans , C. tropicalis , S. cerevisiae , C. glabrata , K. lactis , A. nidulans , N. crassa , A. thaliana and H. sapiens ; the α/β domain, β-barrel, C-terminal arm and SAP domains are highlighted in different colors. (TIF) [file pone.0023732.s001.tif]

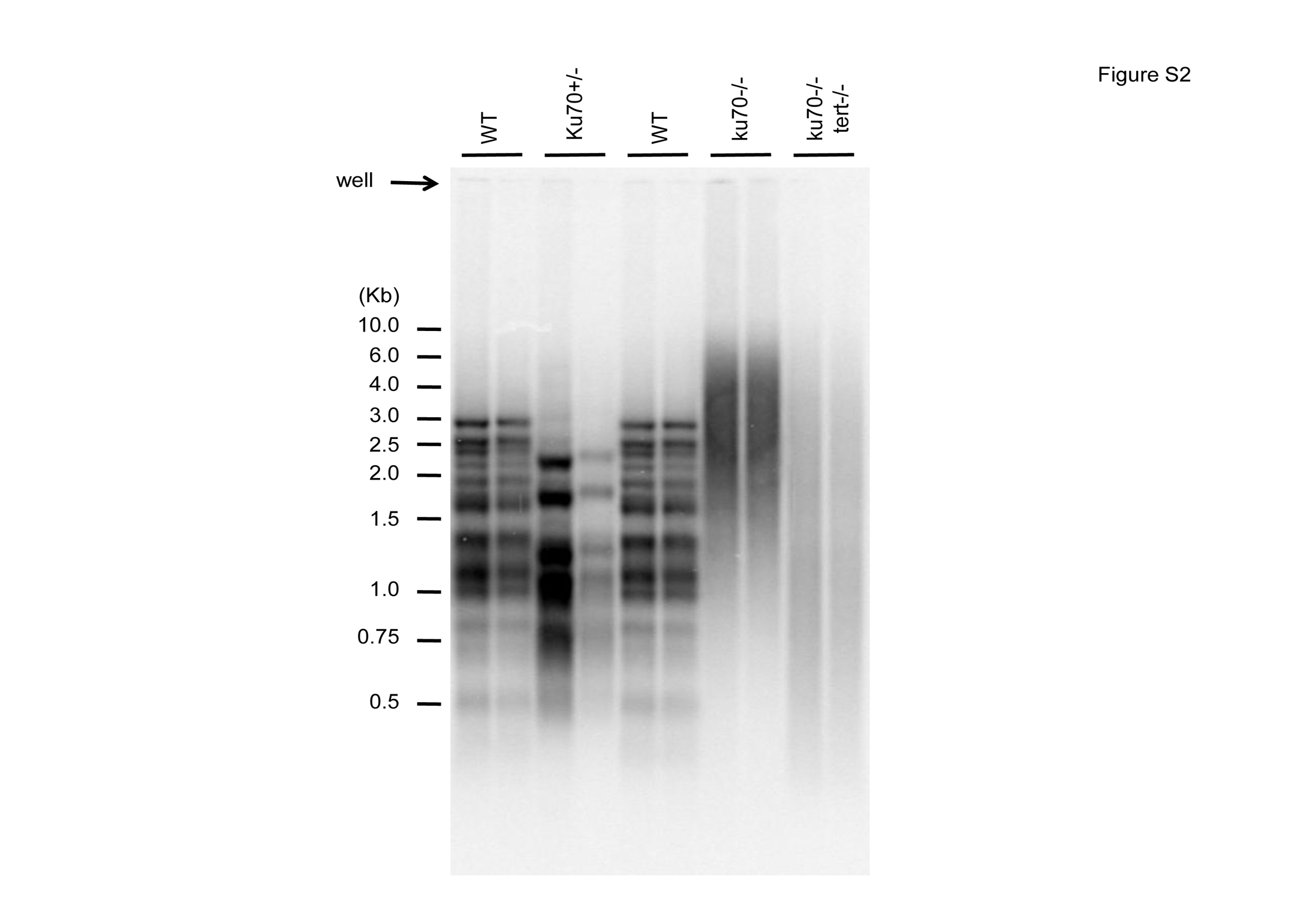

Supplement: Figure S2 — Analysis of telomere lengths in various Ku70 mutants. Duplicate Genomic DNA samples were prepared from the indicated strains following 2 streaks on YPD plates, and subjected to telomere Southern analysis. The entire gel (including the wells) was subjected to transfer and hybridization. The location of the well in the PhosphorImager scan is indicated by an arrow. (TIF) [file pone.0023732.s002.tif]

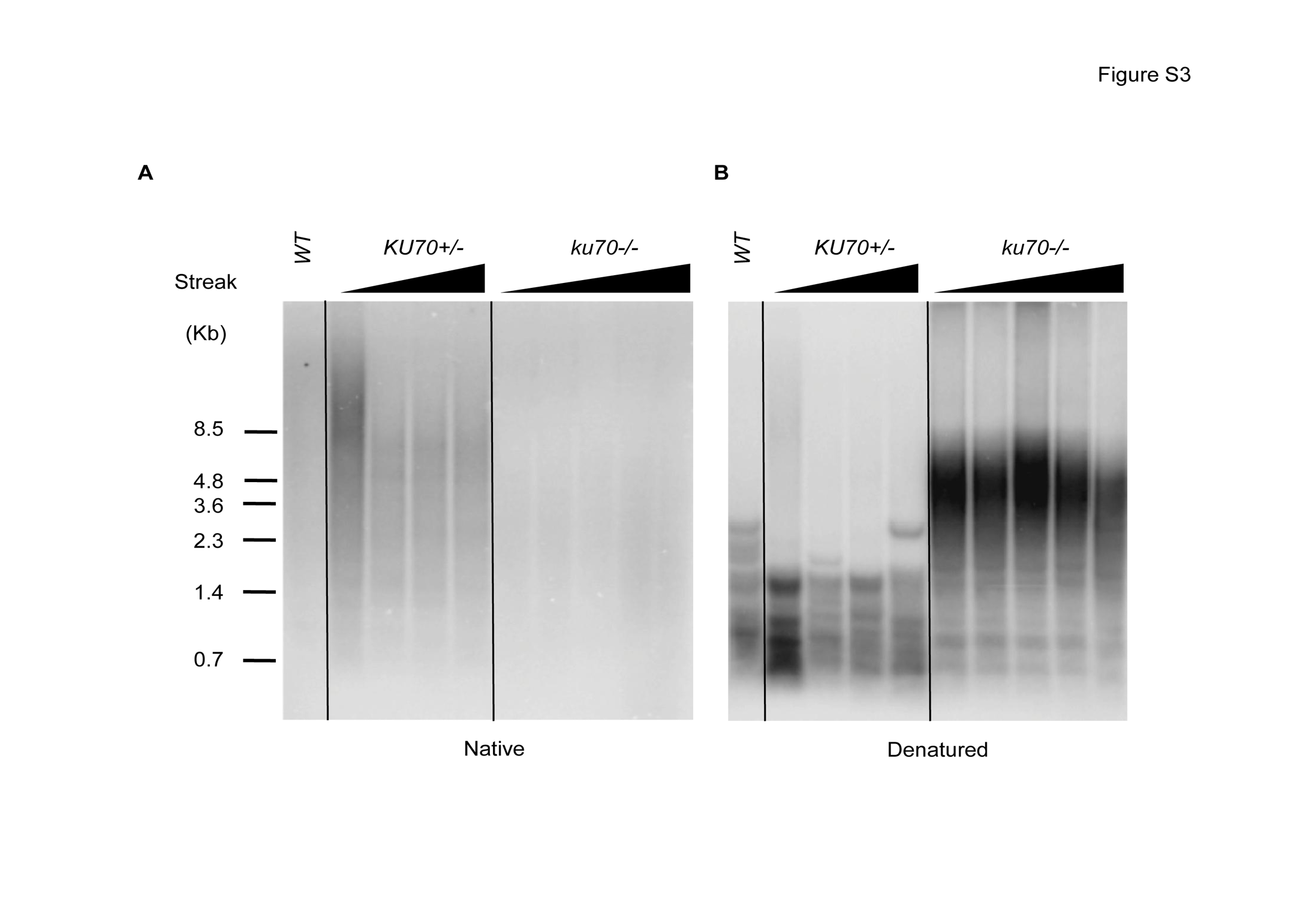

Supplement: Figure S3 — Analysis of G-strand overhangs in the ku70 heterozygous and homozygous mutants. (A) Genomic DNA samples from cultures of the indicated strains were prepared from different passages, and subjected to in-gel hybridization analysis of telomeric G-strand overhangs as described (36). The KU70+/− samples were prepared following 2, 4, 6 and 10 streaks, whereas the ku70-/- samples were prepared following, 2, 4, 6, 8 and 10 streaks on plates. Both the KU70+/− strain (LCD1A.1) and the ku70-/- strain (LCD2A.1) are derived from the CA4 parental strain. (B) After the detection of G-strand overhangs, the DNA fragments in the gel were denatured and hybridized again with the same probe to identify all terminal restriction fragments. (TIF) [file pone.0023732.s003.tif]

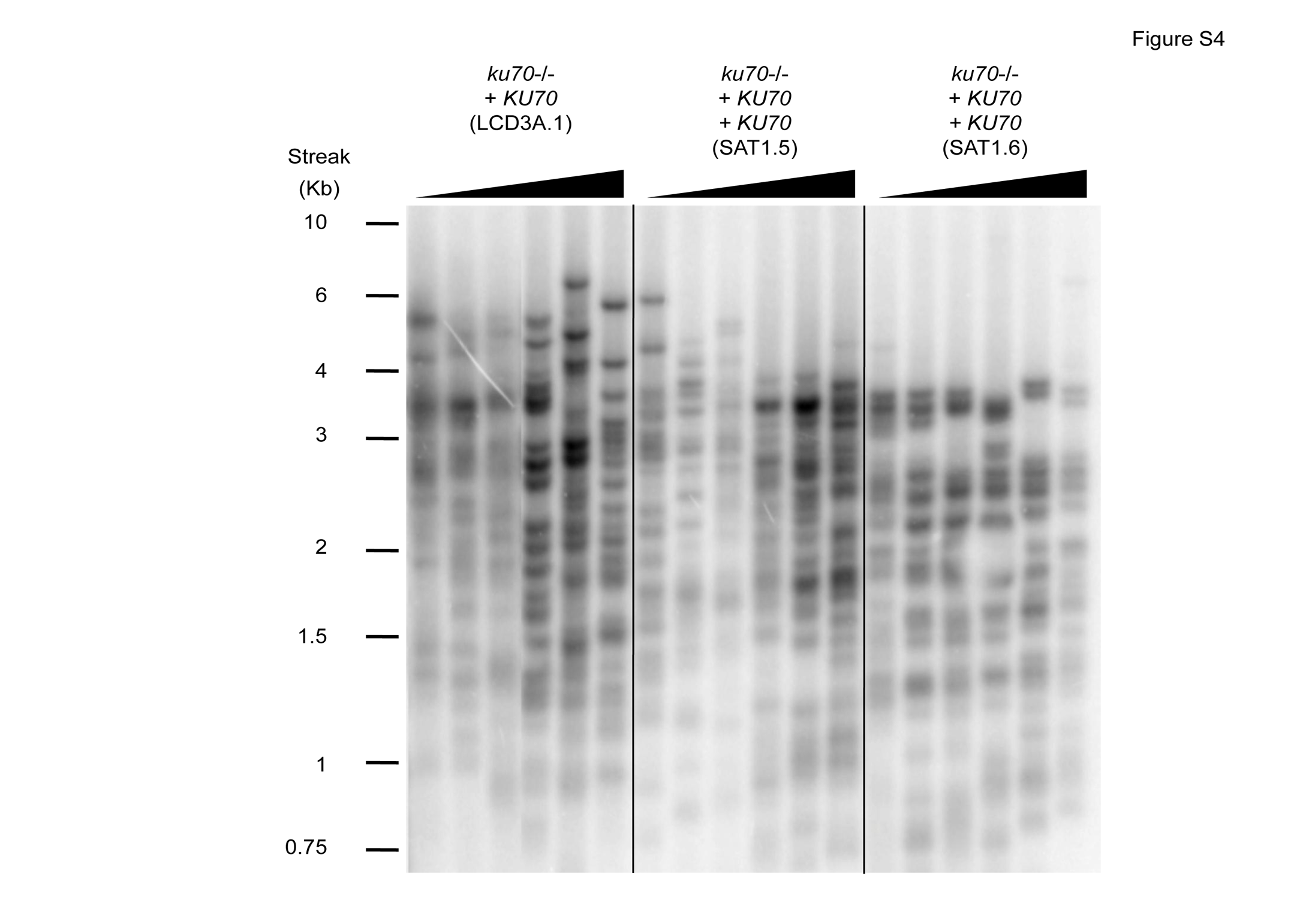

Supplement: Figure S4 — Analysis of the stability of telomere lengths in reconstituted strains. Genomic DNA samples were prepared from the indicated strains following 2, 4, 6, 8, 10 and 12 streaks on YPD plates, and subjected to telomere Southern analysis. (TIF) [file pone.0023732.s004.tif]

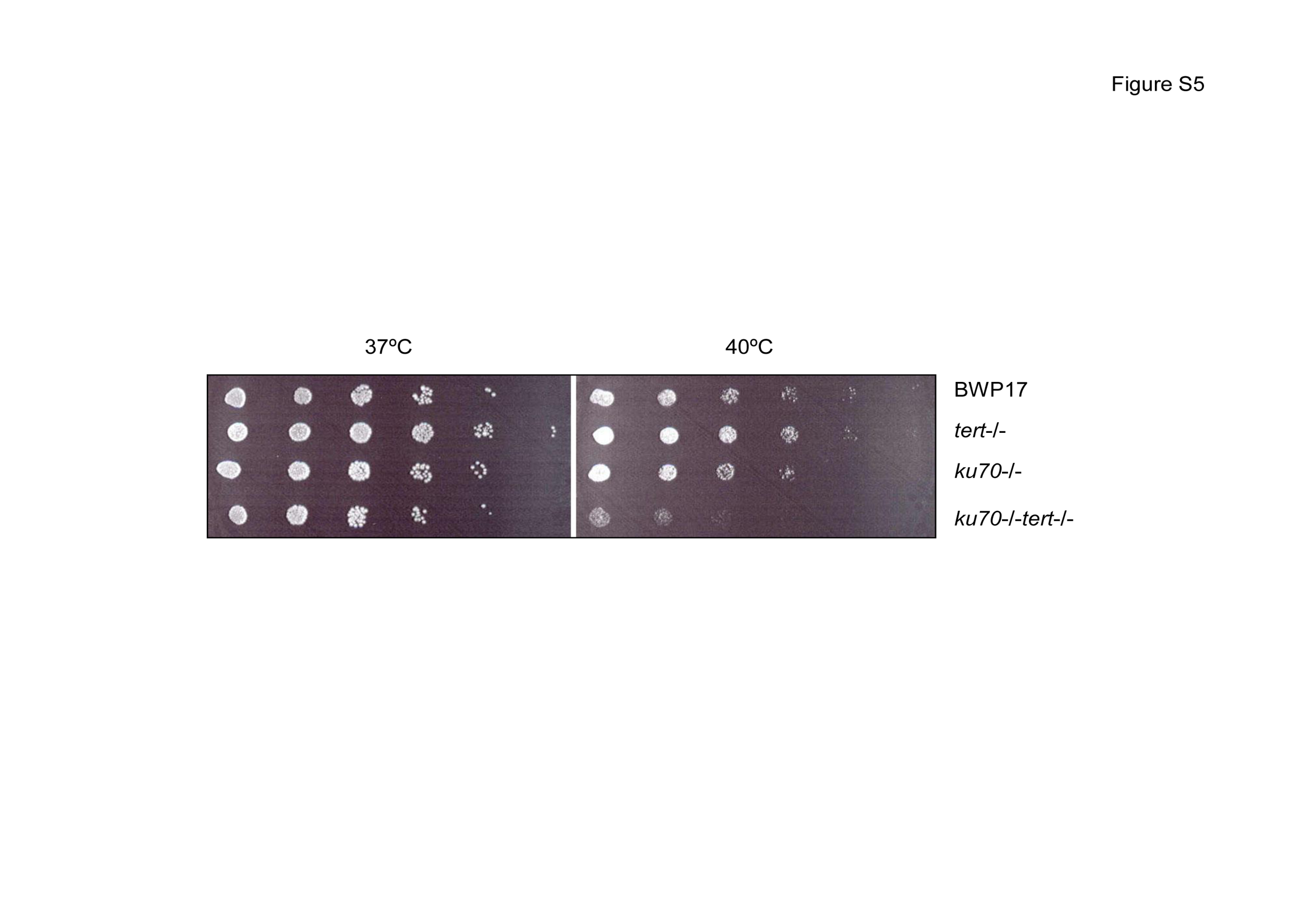

Supplement: Figure S5 — Analysis of the thermosensitivity of telomerase and ku mutants. The indicated strains were tested for thermosensitivity by growing five-fold serial dilutions of each culture on YPD plates at the indicated temperatures. (TIF) [file pone.0023732.s005.tif]
