# Supplementary material for: The Candida albicans Ku70 Modulates Telomere Length and Structure by Regulating Both Telomerase and Recombination
Source: PLoS One. 2011 Aug 23;6(8):e23732. doi: 10.1371/journal.pone.0023732 (PMC3160324; doi:10.1371/journal.pone.0023732)
Supplement: Table S1 — C. albicans , S. cerevisiae , and E. coli strains used in this study. (DOCX) [file pone.0023732.s006.docx]

**Table S1**. ***C. albicans*, *S. cerevisiae* and *E. coli*** **strains used in this study**.

| **Strains** | **Revelant genotype** | **Reference** |
| --- | --- | --- |
| ***C. albicans***  BWP17 | *ura3*Δ::*imm434*/*ura3*Δ::*imm434*,  *his1*Δ::*hisG*/*his1*Δ::*hisG*,  *arg4*Δ::*hisG*/*arg4*Δ::*hisG* | [1] |
| LCF2.1 | *ura3*Δ::*imm434*/*ura3*Δ::*imm434*,  *his1*Δ::*hisG*/*his1*Δ::*hisG*,  *arg4*Δ::*hisG*/*arg4*Δ::*hisG*,  *ku70*Δ::*hisG*/*ku70*Δ::*hisG* | This work |
| *tert* | *ura3*Δ::*imm434*/*ura3*Δ::*imm434*,  *his1*Δ::*hisG*/*his1*Δ::*hisG*,  *arg4*Δ::*hisG*/*arg4*Δ::*hisG*,  *tert*Δ::*hisG*/*tert*Δ::*hisG* | [2] |
| LNL2.1 | *ura3*Δ::*imm434*/*ura3*Δ::*imm434*,  *his1*Δ::*hisG*/*his1*Δ::*hisG*,  *arg4*Δ::*hisG*/*arg4*Δ::*hisG*,  *tert*Δ::*hisG*/*tert*Δ::*hisG*  *ku70*Δ::*hisG*/*ku70*Δ::*hisG* | This work |
| CAF2 | *ura3*Δ::*imm434*/*URA3* | [3] |
| CAI4 | *ura3*Δ::*imm434*/*ura3*Δ::*imm434* | [3] |
| CEA2.5 | *ura3*Δ::*imm434*/*ura3*Δ::*imm434*,  *lig4*Δ::*hisG*/*lig4*Δ::*hisG* | [4] |
| TCR2.1.1 | *ura3*Δ::*imm434*/*ura3*Δ::*imm434*,  *rad52*Δ::*hisG*/*rad52*Δ::*hisG* | [5] |
| LCD1A, LCD1B and LCD1C | *ura3*Δ::*imm434*/*ura3*Δ::*imm434*,  *KU70*/*ku70*Δ::*hisG*-*URA3*-*hisG* | This work |
| LCD1A.1 | *ura3*Δ::*imm434*/*ura3*Δ::*imm434*,  *KU70*/*ku70*Δ::*hisG* | This work |
| LCD3A | *ura3*Δ::*imm434*/*ura3*Δ::*imm434*,  *KU70*/*KU70*::*URA3­*-*hisG* | This work |
| LCD2A | *ura3*Δ::*imm434*/*ura3*Δ::*imm434*,  *ku70*Δ::*hisG*-*URA3*-*hisG*/*ku70*Δ::*hisG* | This work |
| LCD2A.1 | *ura3*Δ::*imm434*/*ura3*Δ::*imm434*,  *ku70*Δ::*hisG*/*ku70*Δ::*hisG* | This work |
| LCD3A.1 | *ura3*Δ::*imm434*/*ura3*Δ::*imm434*,  *ku70*Δ::*hisG*/*KU70*::*URA3­*-*hisG* | This work |
| SAT1.5 and SAT1.6 | *ura3*Δ::*imm434*/*ura3*Δ::*imm434*,  *KU70*::*SAT1*/*KU70*::*URA3­*-*hisG* | This work |
| MLE2.1 | *ura3*Δ::*imm434*/*ura3*Δ::*imm434*,  *lig4*Δ::*hisG*/*lig4*Δ::*hisG*,  *ku70*Δ::*hisG*/*ku70*Δ::*hisG* | This work |
| JLT2.1  ***S. cerevisiae***  BY4741  *ku70*Δ  ***E. coli***  JM109 | *ura3*Δ::*imm434*/*ura3*Δ::*imm434*,  *ku70*Δ::*hisG*/*ku70*Δ::*hisG* *,*  *rad52*Δ::*hisG*/*rad52*Δ::*hisG*  Mat **a**; *his3*Δ1; *leu2*Δ0; *met15*Δ0; *ura3*Δ0  BY4741; Mat **a**; *his3*Δ1; *leu2*Δ0; *met15*Δ0; *ura3*Δ0; *YMR284w*::*kanMX4*  RecA1 endA1 gyrA96 thi hsdR17 (r_k_^-^, m_k_^+^)  supE44A(lac-proAB) relA1  [F’traD36 proAB lacl^q^ ZDM15] λ^-^ | This work |

**References**

1. Wilson RB, Davis D, Mitchell AP (1999) Rapid hypothesis testing with *Candida albicans* through gene disruption with short homology regions. J Bacteriol 181: 1868-1874.
2. Steinberg-Neifach O, Lue NF (2006) Modulation of telomere terminal structure by telomerase components in *Candida albicans*. Nucleic Acids Res 34: 2710-2722.
3. Fonzi WA, Irwin MY (1993) Isogenic strain construction and gene mapping in *Candida albicans*. Genetics 134: 717-728.
4. Andaluz E, Calderone R, Reyes G, Larriba G (2001) Phenotypic analysis and virulence of *Candida albicans* *LIG4* mutants. Infect Immun 69: 137-147.
5. Ciudad T, Andaluz E, Steinberg-Neifach O, Lue NF, Gow NAR. et al. (2004) Homologous recombination in *Candida albicans*: role of CaRad52p in DNA repair, integration of linear DNA fragments and telomere length. Mol Microbiol 53: 1177-1194.
