# Supplementary material for: The Candida albicans Ku70 Modulates Telomere Length and Structure by Regulating Both Telomerase and Recombination
Source: PLoS One. 2011 Aug 23;6(8):e23732. doi: 10.1371/journal.pone.0023732 (PMC3160324; doi:10.1371/journal.pone.0023732)
Supplement: Table S2 — Oligonucleotides used in this study. (DOCX) [file pone.0023732.s007.docx]

**Table S2**. **Oligonucleotides used in this study**.

| **Oligonucleotides used in the amplification and sequencing of KU70 (ORF19.1135)** | | | | |
| --- | --- | --- | --- | --- |
| **Oligonucleotide** | **Sequence of oligonucleotide** | **Relative position within the ORF** | | |
| HDF-AF | AGCGAGCTCTAGAATCTCGACTACTG | -416 | | |
| HDF-BR | CCCAAGCTTCTGCCAAGGTGGGAGATGAA | +2881 | | |
| HDF-1F | CGACTACTGATTAATCGATGAATA | -380 | | |
| HDF-1R | CAGCACCAACAGTGGCATCAATCT | +2188 | | |
| HDF-A2F | GACTTGAAAGTGGGGAAACAATCACA | +433 | | |
| HDF-B2R | TCTCGAGGATCATAATGCAGAAGTTC | +1970 | | |
| HDF-A3F | GGATTTGAGATTGGTGGTGGACAAGA | +1246 | | |
| HDF-B3R | CTAATTCCTGCTTCCTCTTTACGATC | +1241 | | |
| HDF-A4F | TGGAACTTAAACCTTCACAAAGACCA | +2030 | | |
| HDF-B4R | GCGTTTGAGTCTGGGTTTGGGTTTCA | +487 | | |
| HDF-5F | GTACTGGATTTGAACGATTATTTCA | +293 | | |
| HDF-5R | CAGTCCCACCAGCTCCTCCTTCTT | +1432 | | |
| HDF-6F | CGGTGACGATAACAATAATGATGAT | +849 | | |
| HDF-6R | CCAAACCATTCTTCGTTTATTGAA | +597 | | |
| HDF-7F | GCTTCATTGTATCGATCATGTGTGAA | +1618 | | |
| HDF-7R | GCCAACTCATGATAAAGTCAATTGGT | +10 | | |
| **PCR primers for making the *KU70* disruption cassette** | |  | | |
| **Oligonucleotide** | **Sequence of oligonucleotide** |  | **Introduced restriction site** | |
| HDF-AF | AGC***GAGCTC***TAGAATCTCGACTACTG |  | *Sac*I | |
| HDF-AR | GGA***AGATCT***TCCAATCCCAGTACTTCTAG |  | *Bgl*II | |
| HDF-BF | ACG***CGTCGAC***GCCACTGTTGGTGCTGAATG |  | *Sal*I | |
| HDF-BR | CCC***AAGCTT***CTGCCAAGGTGGGAGATGAA |  | *Hind*III | |
| **PCR primers for making ribosomal DNA specific probe** | |  | | |
| **Oligonucleotide** | **Sequence of oligonucleotide** |  | | **Size of PCR product** |
| 5.8Sf | GTTTTTCTTTGAAACTTGCTTTGGCGGTGGGCCCAGCCTGC |  | | 427 bp |
| 5.8Sr | GGTCAAAGTTTGAAGATATACGTGGTGGACGTTACCGCCGCAAGC |  | |  |
| 28sF | GGGACCCGAAAGATGGTGAACTATGCCTGAATAGGGTGAAGCCAG |  | | 1019 bp |
| 28Sr | CTATTCCTTCCTGTGGATTTTCAAGGACCGTCGTAAGCGCACCGG |  | |  |
| 5sF | CTCTGGGGGAGAATACATAGAAAATTTCTTGGCGGGACGACATAG |  | | 1055 bp |
| 5sR | GAGCCCTAAAAGTGAATTTGGTCACGTGAGAGACAGCAGATAAGG |  | |  |
| **Oligonucleotide for telomere Southern** [1] | |  | | |
| CaC2 | ACACCAAGAAGTTAGACATCCGTACACCAAGAAGTTAGACATCCGT |  | | |

**References**

1. McEachern MJ, Hicks JB (1993) Unusually large telomeric repeats in the yeast *Candida albicans*. Mol Cell Biol 13: 551-560.
